# Refractory Crohn’s Disease Responsive to Dietary Therapy: A Case Report

**DOI:** 10.7759/cureus.61262

**Published:** 2024-05-28

**Authors:** Farah Slaczka, Mateusz Slaczka, Ejaz Janjua

**Affiliations:** 1 Medical School, Trinity School of Medicine, Warner Robins, USA; 2 Internal Medicine, University of North Carolina Hospitals, Chapel Hill, USA

**Keywords:** gastroenterology, inflammatory bowel disease, diet therapy, anti-inflammatory diet, crohn’s disease (cd)

## Abstract

Crohn’s disease is a type of inflammatory bowel disease (IBD) that typically presents in the second or third decade of life. There are various pharmaceutical therapies that have been developed to treat the disease's symptoms. However, some patients still do not find relief with these medications and turn to other therapies such as diet modification. The underlying cause of Crohn’s disease involves multiple factors such as uncontrolled inflammation and several genetic variants. While most current medication therapies control the symptoms that occur due to this uncontrolled level of inflammation, an anti-inflammatory diet (AID) may actually lower the level of inflammation in the gut and therefore reduce the amount of disease symptoms in Crohn’s disease. Some such diets include the IBD-AID, Crohn’s disease exclusion diet, and the Groningen AID (GrAID). This report describes a case of treatment-resistant Crohn’s disease in a patient who was given all categories of pharmaceutical therapies including prednisone, budesonide, sulfasalazine, olsalazine, 6-mercaptopurine, methotrexate, mesalamine, and adalimumab. These only gave temporary relief of symptoms and eventually failed for various reasons including allergic reaction, insufficient symptom control, and antibody formation against the medication. This prompted the patient to independently research AIDs instead. In conclusion, for patients whose disease is refractory to different treatments, or who develop antibodies to the medication, AIDs may offer a solution to reduce disease symptoms and progression. Education of healthcare professionals and patients alike is vital in order for Crohn's patients to gain the benefits from dietary therapy.

## Introduction

Crohn’s disease is a type of inflammatory bowel disease (IBD) that manifests as chronic inflammation in any part of the gastrointestinal tract. It has an increasing prevalence worldwide and several factors have been named as contributing to the development of this disease. These factors include genetic susceptibility, environmental triggers, and a dysregulated immune system [1]. It typically presents in younger patients, usually in the second or third decade of life. An analysis of 147 studies published in The Lancet reports the highest prevalence of Crohn’s disease to be in Europe and North America, specifically 322 per 100,000 in Germany and 319 per 100,000 in Canada. It is also reported that 16 of 22 studies on Crohn’s disease reported stable or decreasing incidence of IBD in Europe and North America [2].

Crohn’s disease can be described as having the presence or absence of intestinal complications such as fistula, abscess, or stricture. The disease often progresses from the non-stricturing to stricturing type of disease; however, a large majority of cases already have intestinal complications at the time of diagnosis [3]. 

There are various therapy methods that have been developed with the goal of both disease remission and prevention of disease progression. A treatment plan must be individualized for each patient according to their clinical symptoms, risk stratification, and patient preference. In general, first-line therapy typically consists of steroids to relieve immediate symptoms while anti-tumor necrosis factor-alpha (TNF-α) therapy is started. Other therapies include monoclonal antibodies, immunomodulators, or surgery if necessary [4]. The case presented in this report is of a male patient whose treatment regimen included all of the above at some point. Despite this, his disease continued to progress until he conducted individual research and discovered the importance of diet in disease management.

## Case presentation

**Figure 1:**
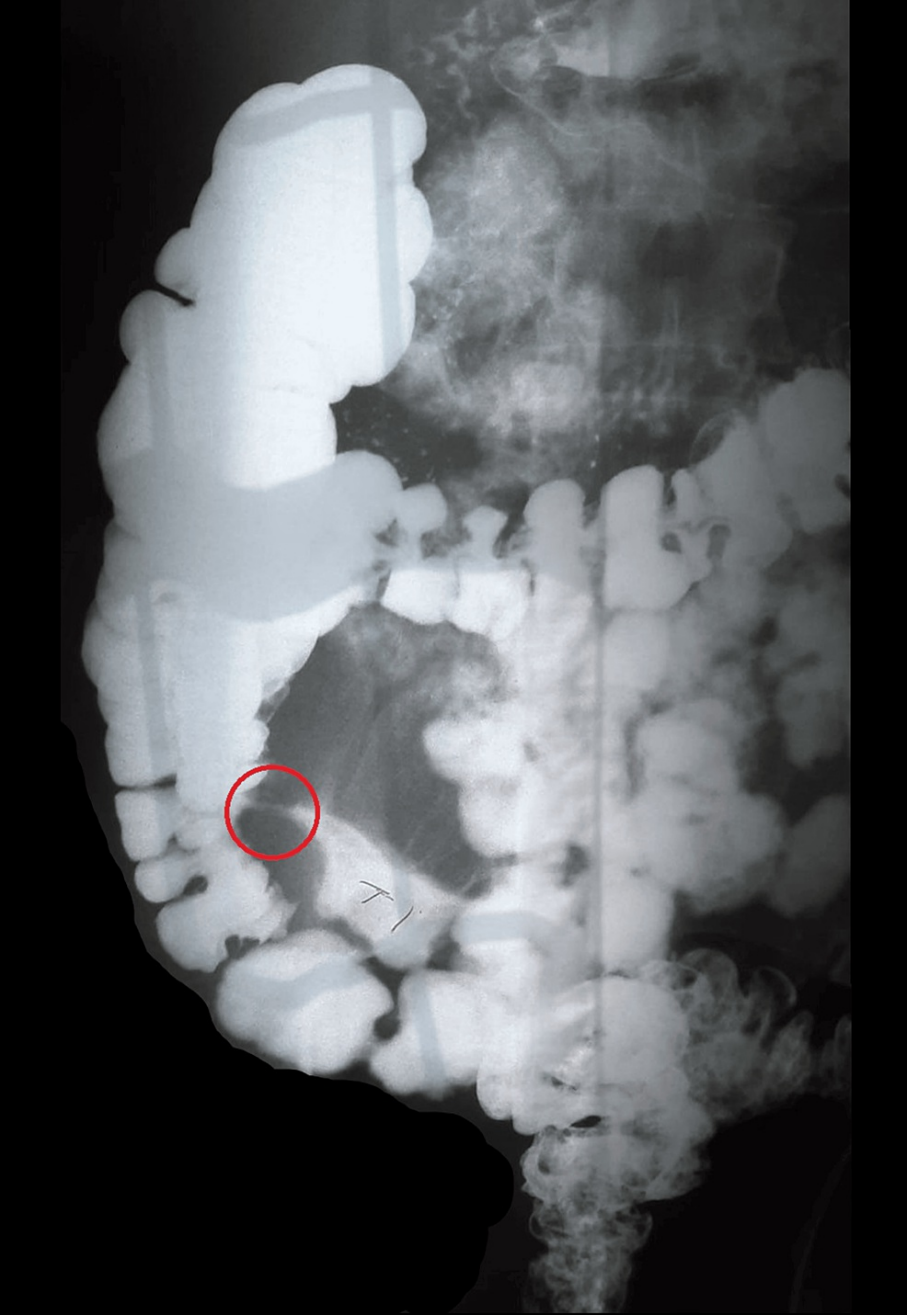
Contrast enema of the colon at the time of initial disease diagnosis showing a stricture (circled) at the terminal ileum

The patient is a 58-year-old male with a long history of Crohn’s disease. He was initially diagnosed in 1985 at the age of 20 during his junior year of college. At that time, he was experiencing increasingly severe pain in the lower right quadrant of the abdomen and he sought medical attention. A barium swallow test was performed and showed a stricture in the terminal ileum, as seen in Figure 1, leading to a diagnosis of Crohn’s disease.

He was initially started on high-dose prednisone 40 mg to get the inflammation under control. The prednisone therapy was slowly tapered down over a few months due to the patient developing cushingoid side effects of weight gain and moon facies. He was then started on sulfasalazine; however, within a few weeks, he developed rashes on his face, lips, and neck, indicating that he had a sulfa allergy. Sulfasalazine was discontinued, and he was again started on prednisone at a low dose.

About six months later, he developed several fistulas leading to abscesses, the worst of which developed on the hip. The hip abscess required surgical debridement and the patient endured a painful recovery period. The patient was continued on prednisone for another few months until his doctor recommended starting Dipentum (olsalazine). This medication seemed to work initially, allowing the prednisone dosage to be tapered down to 5 mg. The patient was stable for several years with occasional flare-ups, which were treated symptomatically with temporary increases in prednisone dosages and a liquid diet. He experienced one very severe flare-up in 1990, which required hospitalization and administration of IV steroids. Over the next few years, he started to have an increased incidence of flare-ups and abdominal pain that eventually became constant, and it was at this point that his doctor recommended surgery.

**Figure 2:**
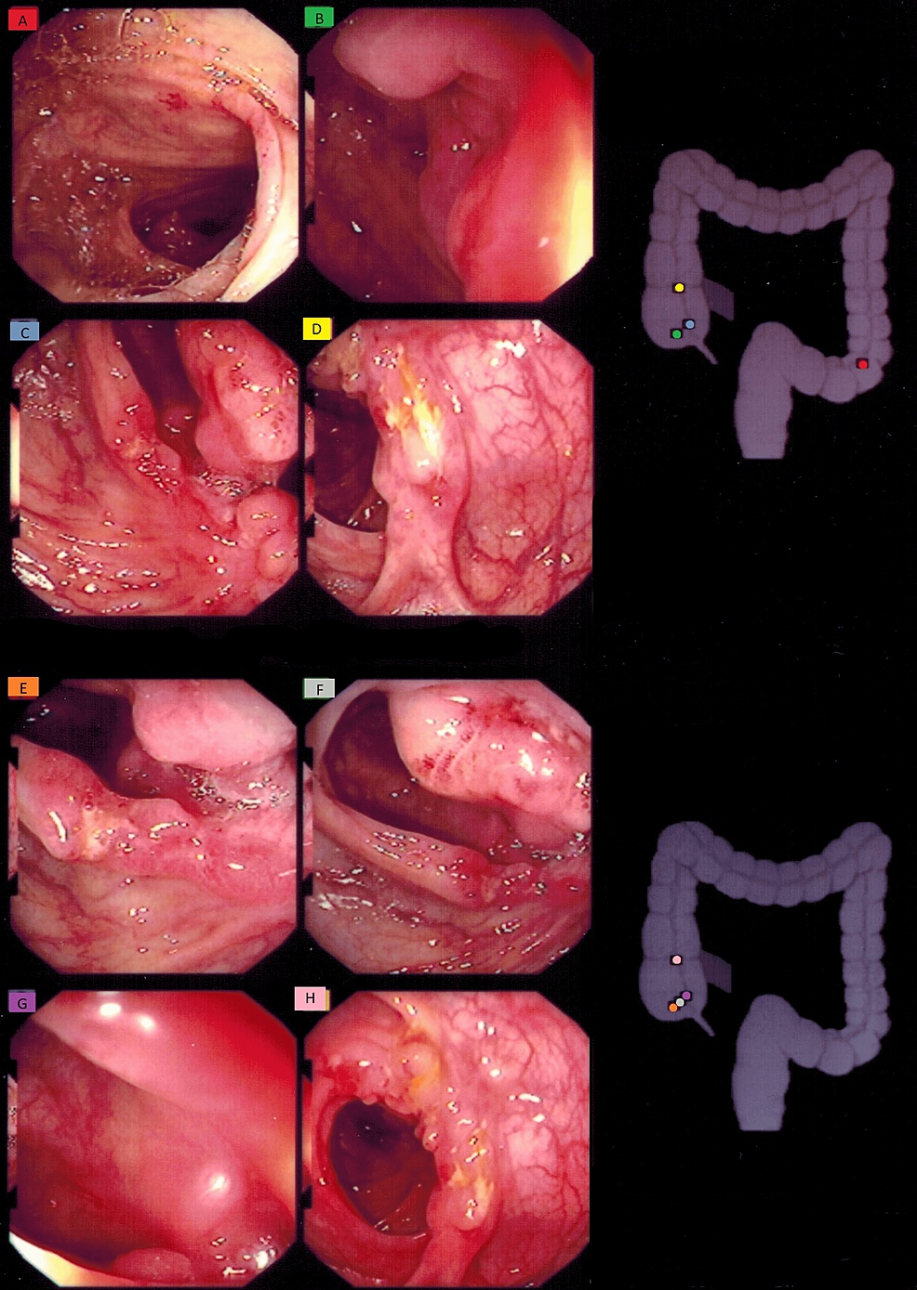
Colonoscopy performed prior to the first ileocecectomy procedure. Image A shows colitis in the sigmoid colon. Images B and C show colitis and nodular tissue around the terminal ileum. Image D shows colitis in the ascending colon. Images E and F show ulcerated folds of mucosa around the ileocecal valve. Image G shows a colon polyp. Image H shows colitis and nodular, ulcerated mucosal tissue in the ascending colon. Pathology report from the ileocecectomy procedure described some pseudopolyp formation and acute inflammation of the lamina propria and luminal mucosa. There is mild crypt distortion without crypt abscess formation yet. There are marked chronic inflammatory cell infiltrates including plasma cells. An area of erosion with acute colitis and a focal area of granulation tissue are also present.

The patient had his first ileocecectomy procedure in 1997. Colonoscopy images prior to this procedure are shown in Figure 2. Three inches of the terminal ileum and 3 inches of the cecum were removed during this surgery. The patient believed his condition to be resolved by this surgery, and therefore did not continue any medications afterward. This led to an increase in abdominal pain again, until it became severe enough for the patient to be restarted on prednisone. His condition continued to go downhill for the next two years until in 2000 the flare-ups became so severe that he described the pain as “bricks moving through his GI tract.” At this point, the doctors recommended a second colon resection.

**Figure 3:**
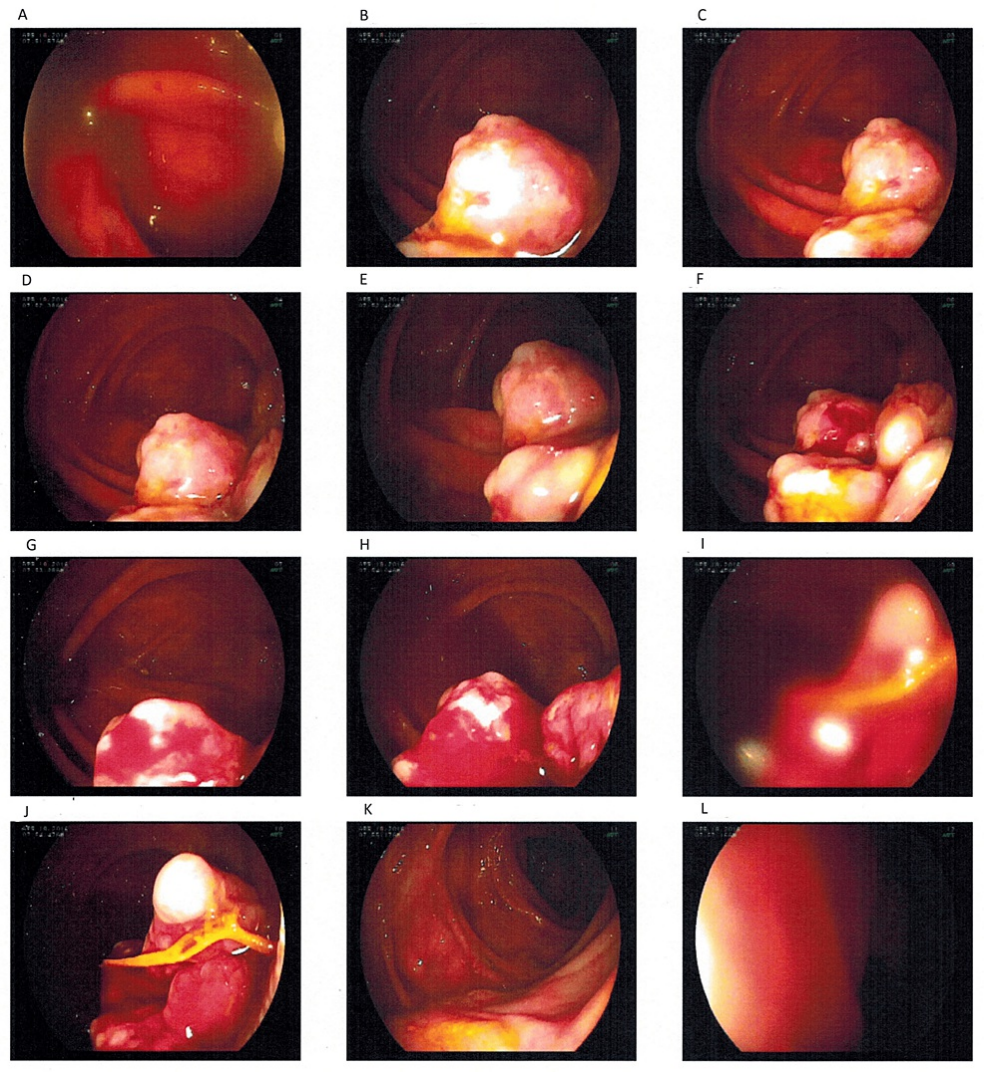
Colonoscopy performed prior to the third ileocecectomy Colonoscopy image A shows the beginning of the anastomosis site from the previous surgeries. Images B-J show masses present at the anastomosis site, which extended to the transverse colon. Image K shows ulcerated mucosa in the transverse colon. Image L is of the descending colon. The pathology report from the ileocecectomy surgery described sections of ileum showing active ileitis with crypt hyperplasia and scattered regions of ulceration. There was also transmural inflammation and granulation tissue with marked inflammatory changes.

After the second surgery, several other medications were attempted such as 6-mercaptopurine and methotrexate, but had to be discontinued due to side effects such as severe reduction in hemoglobin levels. Asacol (mesalamine) was then started, and this drug along with low-dose prednisone was effective for approximately 15 years with no adverse reactions. He continued to have occasional flare-ups which were treated symptomatically. In 2014, the pharmaceutical company that produced Asacol changed the chemical formula into a new drug, Delzicol. This change was suspected to be a possible cause of the increase in symptoms for the next two years, which ultimately led to his third surgery in 2016. Colonoscopy images taken prior to this surgery are shown in Figure 3.

After only two months post surgery, he again began experiencing severe abdominal pain even while on prednisone. At this point, his doctor recommended trying Humera (adalimumab), a biologic anti-TNF immunosuppressant medication. This treatment seemed to work for about two years; however, in 2018 he again began experiencing symptoms. After further workup, it was discovered that his immune system developed antibodies against Humera, nulling the effectiveness of the drug. His doctor recommended trying a different biologic; however, the patient refused due to his high suspicion that it would end in the same result. Unfortunately, due to the patient’s decision to not pursue another biologic, the gastroenterologist said he can no longer remain a patient there. It was at this point that the patient felt there were no other options. His situation was dire due to severe weight loss and he decided to do his own research into his condition. He began tracking what foods caused his abdominal symptoms to worsen and looked into various AIDs.

After a few years of trial and error with various diets and doing copious research into the mechanisms of inflammation, the patient found a diet that allowed him to become completely medication-free, including prednisone, since 2021, and recover his lost weight. His current diet comprises the following: a glass of water first thing in the morning along with a tablespoon of olive oil with a few drops of lemon juice; one pasture-raised egg, organic oatmeal with walnuts and almonds, and an organic banana for breakfast; one organic apple as a mid-morning snack; a rotation of broccoli/potatoes, cabbage, cauliflower/potatoes, or zucchini cooked with organic onions, garlic, and ginger eaten with quinoa and sauerkraut for lunch; one organic banana as an afternoon snack; chicken or ground turkey with organic sweet potato also eaten with quinoa for a second lunch; wild-caught salmon also eaten with quinoa and grass-fed yogurt for dinner; and clove tea before bed. All meals are cooked in coconut oil and contain very mild spices such as sea salt and turmeric, and the patient also takes one capsule of omega-3 fish oil daily. As mentioned earlier, this diet was developed by the patient through an elimination process and individual research into anti-inflammatory foods and was a mixture of various other established AIDs. 

## Discussion

The pathogenesis of Crohn’s disease involves an unrestrained immune response to luminal gut antigens. Several genetic variants have been associated with a higher likelihood of developing Crohn’s. Mutations in the *Muc2* gene result in reduced intestinal mucus production, and mutations in the *FUT2* gene cause a reduction in the secretion of ABO antigens resulting in alterations of bacterial interaction or adhesion with these antigens or mucus, leading to an increased risk of developing Crohn’s disease [5]. Another mechanism that is associated with making a person more prone to developing this disease is a higher production of cytokines such as interleukin-12 (IL-12), interferon-gamma (IFN-γ), and TNF-α by T cells and macrophages [5]. These cytokines may be a contributing factor to the increased immune response in these patients, leading to worsening inflammation in the bowel eventually resulting in Crohn’s disease or ulcerative colitis.

With regards to disease management, corticosteroids have long been known to effectively reduce levels of inflammation in Crohn’s disease and ulcerative colitis. However, these medications have numerous unwanted side effects such as cushingoid appearance, osteonecrosis, easy bruising, weight gain, and gastrointestinal disturbance such as dyspepsia. Long-term use in particular puts the patient at risk for infection, impaired wound healing, and steroid dependence. 5-aminosalicylic acid compounds such as sulfasalazine are a mainstay of treatment for ulcerative colitis; however, they have shown little if any benefit for Crohn’s patients, despite their extensive use [6]. In the case of the current patient, discontinuation of sulfasalazine was necessary due to an allergic reaction. Thiopurines such as 6-mercaptopurine act as antimetabolites and immunomodulators, and allow treatment to occur without the use of steroids. However, they have a slow onset of action, requiring them to be used for long-term, rather than short-term, therapy. Side effects of this class of drugs include pancreatitis, hepatotoxicity, and myelosuppression [6], which is likely what this patient experienced resulting in his dangerously low hemoglobin levels.

The most effective drug in the current patient’s disease history was Asacol (mesalamine). There are conflicting reports about the efficacy of this drug in the remission of Crohn’s patients, with one study showing 25% relapse in patients on a trial of mesalamine [7]. However, for this patient, mesalamine kept him steady for a period of 15 years before his condition again went downhill, possibly due to the change in drug formulation by the pharmaceutical company. The newer type of treatment used for this patient was Humira (adalimumab), which is a type of anti-TNF-α that is self-injectable subcutaneously. The effectiveness of this therapy was cut short due to the patient’s development of auto-antibodies against the drug. This is a rather rare occurrence, with one study citing only one out of 225 patients developing antibodies against adalimumab, although this number is likely an underestimation due to the short study duration [6]. The development of antibodies in this patient highlights his unique disease presentation and the difficulty involved with controlling his Crohn’s disease.

The eventual result of all these failed medication regimens was the patient pursuing alternative methods of controlling his disease, specifically through AIDs. An article published by Harvard University acknowledges the fact that permanent reduction of inflammation in certain autoimmune conditions can be achieved not through pharmaceuticals, but rather through appropriate AIDs and avoidance of foods that can trigger inflammation. Some foods that are known provokers of inflammation include refined carbohydrates (white bread), fried foods, sweetened beverages, and processed meats [8]. Such foods are often also blamed for the development of other diseases such as diabetes, obesity, and heart disease, likely due to their inflammatory qualities. Therefore, it is appropriate to advise patients in general to avoid such foods, particularly if they are at higher risk for developing autoimmune or other health conditions. On the other hand, foods that contain anti-inflammatory qualities include fatty fish (salmon), nuts, green leafy vegetables, olive oil, and fruits [8]. In the case of this patient, he came to a similar conclusion about which foods to include in his diet and which ones to avoid, which led him to his current diet which was described earlier. In addition to the types of foods, it is also essential to pick organic and natural ingredients as much as possible, as this further reduces the risk of ingesting potentially inflammatory chemicals or preservatives. Prebiotic and probiotic foods have also been demonstrated to show significant positive effects in reducing Crohn’s symptoms in this patient, and studies have been done to assess their effects on the intestinal microbiome. Prebiotic foods act as food for human gut microflora and include foods such as onions, garlic, bananas, and whole grains. Probiotic foods contain live microorganisms to maintain healthy normal gut flora and include yogurt, kefir, and fermented foods such as sauerkraut and pickles [9]. The patient started adding prebiotic fiber from organic vegetables, and it took about two years after adding prebiotics to his AID for his symptoms to resolve enough for him to stop all medications. After stopping all medications, he added probiotic foods like sauerkraut and yogurt which provided additional symptom relief after a few months.

**Figure 4:**
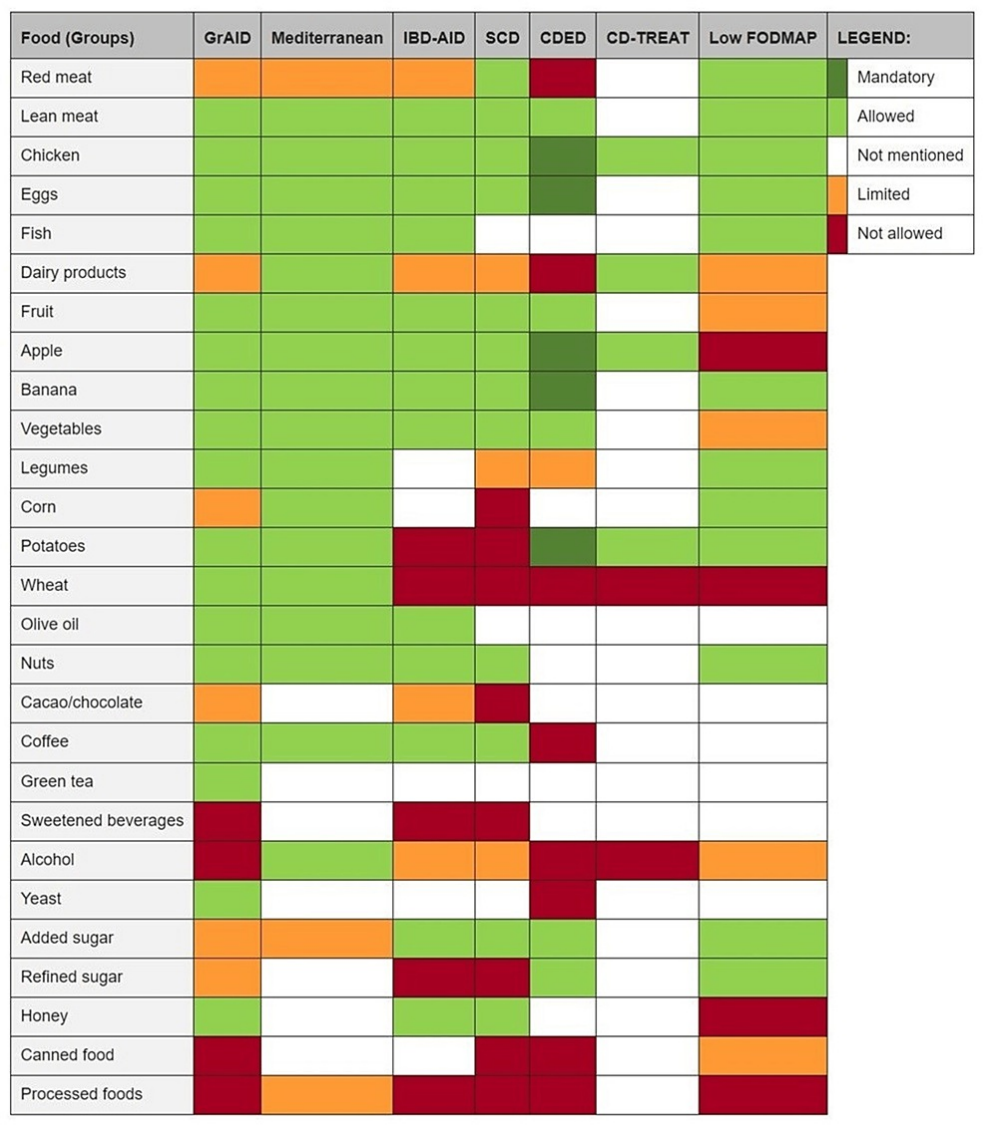
Overview of GrAID compared to diets being tested in inflammatory bowel disease with mandatory, allowed, limited, and not allowed food (groups) Copyright/license: This figure has been adapted from Campmans-Kuijpers and Dijkstra, 2021 [10], an open-source article distributed under the terms and conditions of the Creative Commons CC BY license. GrAID, Groningen anti-inflammatory diet; IBD-AID, inflammatory bowel disease anti-inflammatory diet; SCD, specific carbohydrate diet; CDED, Crohn's disease exclusion diet; CD-TREAT, Crohn's disease treatment-with-eating diet; Low FODMAP, low-fermentable oligosaccharide, disaccharide, monosaccharide and polyols diet.

Several studies have been done on various diets created for IBD patients in an attempt to control their symptoms, particularly if medications were insufficient or ineffective. One such study observed the effect of the IBD-AID diet, which restricts the intake of certain carbohydrates and includes pre- and probiotic foods along with dietary fatty acids. The results of this study showed some improvement in IBD symptoms in these patients [10]. Additionally, another study showed that partial enteral nutrition combined with a Crohn’s disease exclusion diet (CDED) was a useful therapy method in Crohn’s patients who failed biologic therapy [10]. Based on the data from past trials with various diets, a group of researchers at the University Medical Center Groningen in the Netherlands compiled the information to create the GrAID diet (Groningen AID), which they hope can be used as a sort of guideline for IBD patients when trying to determine what they should eat. The researchers compared the GrAID diet recommendations to several other diets and compiled their data as shown in Figure 4 [10].

The GrAID diet seems to be in general agreement with the diet discovered by the patient in this case report, by recommending the intake of lean protein, high-fiber vegetables and fruits, and fish. Such a diet appears to increase healthy gut microbiota and decrease the level of inflammation in Crohn’s patients, which in turn reduces the frequency and severity of symptoms.

The anti-inflammatory effects of the foods contained in these diets have been well-researched. One paper discusses the benefits of omega-3 and its derivatives in reducing inflammation. The omega-3 derivative known as eicosapentaenoic acid (EPA) can be metabolized by the same enzymes as arachidonic acid (AA), which is normally involved with the formation of inflammatory markers via the COX and LOX pathways. This means that if there is a higher concentration of EPA in the body, it can occupy these enzymes in a greater quantity than AA, thus reducing the formation of COX and LOX inflammatory markers [11]. Another paper describes the benefits of phenolic acids, which are found in various fruits and vegetables. The phenolic groups in polyphenols can accept an electron to form stable phenoxyl radicals which disrupts chain oxidation reactions in cellular components. Additionally, polyphenol-rich foods and beverages may increase plasma antioxidant capacity, leading to a reduction in oxidative damage to cellular DNA which can usually stimulate an inflammatory response [12]. These are just two examples of how anti-inflammatory foods can reduce inflammation at the cellular level. 

## Conclusions

IBDs such as Crohn’s disease can affect many aspects of a patient’s life. It requires them to be hyper-aware of their diet and their environment due to symptoms of frequent bowel movements and abdominal pain. This disease may even be the reason some patients avoid participating in activities that they used to find enjoyable. It is therefore essential for this disease to be controlled as much as possible not only to benefit the patients’ physical health but also their emotional well-being. There have been many advances in therapeutic regimens to treat this condition over the past several decades, some of which include steroids, TNF-α-inhibitors, and biologicals. While many patients do benefit from these treatments, some patients continue to experience symptoms and are refractory to therapeutic treatments.

For such patients with resistant disease, AIDs may be the solution for them to control their symptoms and also eventually permanently control the level of inflammation occurring in their gastrointestinal tract. The patient in this case report found relief of symptoms through trial and error of various anti-inflammatory foods, probiotics, and prebiotics. However, current dietary guidelines for IBD patients are lacking. The role of diet in the IBD disease process is not usually emphasized to patients, as is demonstrated by the current patient, who had to conduct his own research to discover the ability to control disease symptoms through diet. Therefore, healthcare providers should be encouraged to also educate themselves on this topic so they may in turn help educate their Crohn’s patients as well. This is important not only for patients whose disease becomes refractory to therapeutic treatment but also for patients earlier on in their disease history to prevent the progressive severity of their disease symptoms by controlling levels of inflammation from the start.
